# The juggling act of pharmacists in Sweden: a qualitative study on balancing healthcare professionalism and retail employment

**DOI:** 10.1186/s12913-024-11682-w

**Published:** 2024-10-10

**Authors:** Kenneth Hagsten, Andrea Eriksson, Ingrid Svensson, Kristina Palm

**Affiliations:** 1 Karolinska Institutet, Solna, Sweden; 2 KTH Royal Institute of Technology, Huddinge, Sweden; 3 Karlstad University, Karlstad, Sweden

## Abstract

**Background:**

Community pharmacies in Sweden merge a state-funded professional community service with a retail business. While previous research has recognized its challenges, less attention has been paid to the potential conflict of interest it could result in regarding the work of pharmacists. This study aims to increase understanding of how pharmacists in Sweden combine their healthcare ambitions and obligations with those concerning being employees in a retail business.

**Methods:**

Semi-structured interviews were conducted with 28 pharmacists employed by pharmacy chains in Sweden. The data were thematically analysed.

**Results:**

In the analysis, three themes were formulated: Different interests, The conflict between professional work & business work, and Strategies to manage the conflicts. The analysis of the data reveals that pharmacists in Sweden experience a conflict of interest. They grapple with balancing being healthcare professionals with a focus on patient care and being valued retail employees that also focus on financial results. This conflict manifests in various situations, such as time management, communication focus, preferred behaviours, and different tasks. The different strategies applied by individual pharmacists to manage the work are results of job crafting. Pharmacists employ strategies such as compromise, mandate stretching, avoidance, and acceptance to manage the conflict. However, these strategies lead to compromises in their work.

**Conclusion:**

This study aimed to understand how pharmacists combine their healthcare ambitions and obligations with those of being employees in a retail business. The study demonstrated that Swedish community pharmacists need to perform a juggling act to be both professional healthcare workers and viewed as valuable retail employees. In situations when the conflict of interest occurs, the pharmacists use different strategies when determining how to prioritize between the professional work and the business work. The conflict of interest in the pharmacists’ work need to be taken into consideration when discussing pharmacists practices, the profession, or role it’s in the healthcare system.

## Background

Compared to most other forms of commerce, a distinctive feature of pharmacy retail in Sweden is the combination of state-funded community service and a retail business. Despite the deregulation of the pharmacy market in 2009, which allowed private businesses to enter the market, it remains highly regulated, necessitating retail permits, licensed staff, pricing restrictions, and supply requirements [1]. Concurrently, the pharmacy industry adheres to the rules of retail business, where companies traditionally compete for customers based on “price, selection, convenience, customer service, and/or the shopping experience” [2].

There are differences between pharmacies around the world, but the literature suggests that European community pharmacies do not differ very much, especially concerning the way they are regulated and the products offered [3]. The main difference seems to be regarding the services available in the pharmacies and the reimbursement of them [3]. The services most common in pharmacies in Martins et al.‘s study, apart from dispensing, were smoking cessation and drug waste management programs. Prescribing, needle exchange programs, and administration of injectable drugs were examples of other services that some pharmacies in Europe offered, but not in Sweden. With the combination of healthcare and retail business, previous research has shown how this can lead to challenges. Vogler et al. [4] stated that an increased focus on profit and sales had been observed in all nine European countries that were included in the study and that economic constraints could increase the workload for pharmacists and compromise the quality of the work when the focus shifts to commercial aims at the expense of public health. A pattern among deregulated pharmacy markets was that businesses put pressure on pharmacists to emphasise financial outcomes at the expense of healthcare work [4]. Kellar et al. [5] describe how pharmacists’ identity primarily is based on activities concerning filling prescriptions and counselling, while the business side of the work is viewed as more undesirable and even in conflict with professional work. The discussion about the expectations of community pharmacists to both be professional and business-oriented is not new; Quinney [6] problematised it already in an article in 1963 and argued that pharmacists’ tendency to violate prescription regulation was based on their orientation in the professional-business spectrum. A UK-based study from 2014 showed that more time was spent on counselling about non-prescribed products compared to prescribed ones [7]. Although the above-mentioned studies explored challenges arising in the combination of healthcare and retail business, the experiences of employed pharmacists were not the focus of these studies.

The picture of the Swedish pharmacy market and pharmacy work is similar to the above-presented international picture. Swedish pharmacies could be characterised as ‘in-between’ healthcare and business, and consequently, pharmacists should both provide advice and sell [8]. Pharmacists in Sweden experienced difficulty in combining professional work with the organization’s expectations of selling additional products and tended to be more loyal to the customers and their profession than towards the overall goals of the organization [9]. Wisell and Sporrong [10] found that Swedish pharmacists felt their professional expertise was underused at pharmacies, and particularly that the practice of counselling had waned after the deregulation of the pharmacy market. Olsson et al. [11] reached a similar conclusion, stating that the dialogue in Swedish pharmacies was not concentrated on enhancing medication utilization; instead, a substantial amount of time was spent on other more administrative issues. Specifically, approximately 4 min on average were spent with each customer, and about 10% of that time was used for communication about pharmaceutical or medical issues [11]. Wisell and Sporrong [10] also show that some pharmacists expressed negativity towards the selling of merchandise as part of their work, as they believed this could not be combined with their professional role in the healthcare sector. Some authors take it further and even question if healthcare combined with commercial retail is the best model for pharmacies [12, 13]. Previous research has highlighted the existence of different perspectives and expectations of pharmacists [4–6, 9, 10]. It seems like the pharmacists have to handle the tension individually, using their experience, common sense, and judgment, but a deeper understanding of this conflict, including how it is managed, is needed [13].

The conflict pharmacists experience in their work could be conceptualized as a ‘conflict of interest,’ based on the definition by Komasoroff et al. [14]. The pharmacists’ perception of their work will likely shape how pharmacists perform their duties. Previous studies have pointed out that it is largely up to the individual employee to manage their work (e.g., [8, 11, 12]), which suggests that they can form, or craft, the execution of the work based on the conflict as well. Job crafting, as described by Tims & Bakker [15] and Wrzesniewski & Dutton [16], is used to theoretically explore the strategies pharmacists use to manage the conflict of interest (CoI) they experience.

While previous research has acknowledged challenges in combining healthcare work and retail business work in pharmacies, to our knowledge, how this conflict between what we label professional work (PW) and business work (BW) is actually managed by employed pharmacists has not been studied.

Therefore, this study aimed to increase the understanding of how pharmacists in Sweden combine their obligations and ambitions as healthcare professionals and as employees in a retail business. To do so, our objectives were to:

- Understand how pharmacists perceive their ambitions and obligations regarding work.
- Understand the conflict of interest that could occur between being a healthcare professional and a retail employee.
- Identify what strategies pharmacists apply to manage situations where a conflict of interest exists.

## The setting

The Swedish pharmacy market consists of 1,407 pharmacies, predominantly operated by nationwide chains, with 48 being independently owned [17]. A store manager oversees the pharmacy, and several pharmacists are typically employed at each site. In Sweden, a government-issued license is required to practice as a pharmacist, obtainable through either a bachelor’s or a master’s program in pharmacology; both qualifications confer the same authority within a pharmacy [18]. Patients may choose any pharmacy to fill their prescriptions. Prescription medications, with some exceptions, are included in the government’s high-cost protection scheme, which subsidizes the patient’s costs up to a threshold, beyond which medications are provided free of charge. Pharmacies are compensated for their work through a Pharmacy Margin on each prescribed product, consisting of a preset fee and a margin [18]. The cost of over-the-counter medicines and other products is borne by the patient, with prices set by the pharmacies. In Sweden, pharmacists themselves must provide individual information and counselling regarding the prescribed medicine and its use, as well as in the dispensing process, “perform other tasks that are of particular importance for the safe handling and use of the medical product” [1].

## Theoretical frame of reference

### Conflict of interest

Thompson [19] describes a CoI as “a set of conditions in which professional judgment concerning a primary interest tends to be unduly influenced by a secondary interest.” Thompson uses the welfare of the patient as one example of a primary interest based on the professional duties of a physician, and financial gain as a secondary interest. Komesaroff et al. [14] further developed the concept of CoI and the importance of the interest, which they define as “a commitment, goal, duty, or obligation related to a specific social role or practice.” A conflict of interest arises when at least two interests coexist and are incompatible with each other in terms of decision-making and action [14]. According to Lipworth [20] the interests, or the desires or obligations as the author describes them, can be non-financial or financial. A non-financial desire could be the ambition for safety, promotion, or increased professional status, while a financial desire is about material means [20]. Furthermore, Lipworth [20] states that the obligations can, for example, be to society, an employer, or patients. The presence of different interests (or different desires and obligations) are a part of life, according to Lipworth, it is when they are incompatible that the problem arises. When determining if a non-financial interest is present, Komesaroff [14] describes three criteria that can be used:

- Pertinence: the interest must be applicable to the issue being considered.
- Substantiality: the interest must generate substantive outcomes in the specific context.
- Immutability: the interest itself and/or the outcomes it generates are not readily susceptible to change because of conversation, dialogue, or reflective deliberation.

Thus, in relation to pharmacies, we understand a pharmacist may face a CoI when in a situation having to choose actions or decisions that will satisfy either the professional work or the business work.

### Job crafting

Job crafting has been defined as a bottom-up process, where employees shape and redesign work in ways that suit their own needs and individual goals [16]. It has been described as actions and activities initiated by the employees themselves; thus, job crafting behaviour/activities may not be sanctioned by formal management [21]. Job crafting has furthermore been described as employees’ strategies to optimize available work resources to be able to deal with demands in their work [15]. Wrzesniewski & Dutton [16] include in their definition of job crafting three dimensions: task crafting, relational crafting, and cognitive crafting. Task crafting includes adding, changing, or expanding job tasks, for example, not prioritizing work assignments that are not in line with professional values. Examples of relational crafting include developing good relations with colleagues and managers or initiating social activities at work. Finally, cognitive crafting embraces employees’ mental strategies of making work feel meaningful [16]. Studies have demonstrated that employees’ opportunities for job crafting strategies, which allow them to integrate their motivations and desires into their work performance, contribute to improved work performance, job satisfaction, job engagement, and the intention to stay within the work organization [15, 22–25].

## Method

To fulfil the explorative aim of increasing the understanding of how pharmacists in Sweden combine their healthcare desires and obligations with those concerning being employees in a retail company, a qualitative study design was employed, comprising semi-structured interviews with pharmacists employed in community pharmacies. The study was approved by the Swedish Ethical Review Authority (2023-00950-01).

### Data and data collection

Data consist of interviews that were conducted over two distinct periods. The initial round was part of a study focused broadly on daily work in pharmacies (see [9]), while the subsequent round concentrated on various aspects of the studied CoI. All interviews were conducted by the lead author, KH (a male PhD student). During the first data collection phase, KH was employed at the pharmacy’s head office, working on internal training and education concurrently with his PhD studies. In the second phase, KH was employed by a university. KH assumed primary responsibility for data collection, analysis, and manuscript drafting. KP (a female Professor with a PhD) and AE (a female Professor with a DrPH) provided supervision and support in the study’s design and both preliminary and final data analyses. IS (a female Post-doctoral faculty member with a PhD) contributed to the final data analysis. All authors were involved in drafting and finalizing the manuscript.

The initial interviews, conducted between 2019 and 2020, involved pharmacists from five different workplaces, all part of the same pharmacy chain. These pharmacies were selected for stability and effective operations, based on key performance metrics such as sales, productivity, leadership, and employee indices. In addition, the participating pharmacies were among the chains top quarter regarding financial turnover, to ensure that they had a sizable workforce where the informants s could be picked for participation using randomization. The aim was to study pharmacists’ work situations with minimal data interference from factors such as unstable results, temporary local challenges, or a limited number of colleagues. Two pharmacists from each pharmacy were randomly chosen for participation. Additionally, the pharmacy manager was included if they, besides managing the pharmacy, primarily performed pharmacist duties. Initial contact with the pharmacies was made via email, providing details about the study, the nature of the questions, the intended use of the results, consent procedures, and the option to withdraw at any stage. Information about the researcher, their affiliations, and the study’s objectives was also shared. A semi-structured interview guide was utilized (see Attachment 1), focusing on understanding the nature of work in pharmacies, including tasks, perceptions of work, workplace relationships, and management of work and responsibilities. Interviews were conducted in Swedish at the informants’ workplaces following the signing of consent forms. When the first set of interview data was analysed, the notion of a conflict of interest in the work became evident. The authors decided to deepen the understanding of these conflicts by conducting a second round of data collection, focused on further understanding this aspect of the work.

Additional interviews were carried out in 2023 to deepen the understanding of CoI among pharmacists. A purposive selection strategy was employed to include informants from diverse age groups, educational backgrounds (e.g., Master of Science or Bachelor of Science in Pharmacy), and genders. All informants were required to have at least one year of experience in a community pharmacy. Social media was utilized to disseminate information and a link for expressing interest in participation. Study details and the participation link were posted in Facebook groups targeted at pharmacists and on the Swedish Pharmacists’ Association website. The final group of informants received comprehensive information about the study, including its objectives, data handling procedures, and the option to opt-out at any time. An interview guide was developed, informed by the theory of CoI to support semi-structured interviews. Questions exploring the expectations and ambitions related to pharmacists’ work from both the pharmacists’ and employers’ perspectives, how these expectations could be met in daily work, challenges in reconciling these expectations, and strategies for managing such difficulties. Due to the explorative nature of the study, the sample size was guided by the concept of information power [26]. By this, specific informants belived to have insights into the objevtives of the study were recruited. Interviews were conducted digitally via Microsoft Teams, with consent obtained before each session. Both the in-person and the interviews conducted via Teams were conducted in Swedish, recorded with a portable voice recorder, and verbatim transcribed either by a third-party vendor or by the first author. The transcript from the first data set was assigned numbers from 1 to 14, and the second set from 15 to 28. The interviews lasted between 30 min and 90 min. 

### Data analysis

When the data collection was finalized, the transcripts from both data sets were thematically analysed together following the phases described by Braun and Clarke [27]. The analysis was made using an inductive approach. The first step of familiarization was concluded when the transcripts were produced and read multiple times. This was succeeded by an initial coding process, where a selection of transcripts was read and coded independently by KH, KP, and AE, followed by a joint workshop. In the workshop, the data and coding were discussed, and categories and beta themes (or candidate themes as Braun et al. [28] refer to these early-stage themes) were identified. KH, KP, and AE found similarities to the job crafting literature during this work and therefore decided to use that in the following analysis, together with CoI. In the second step, the first author read and coded the remaining transcripts, adding new codes when necessary. Thereafter, all authors reviewed and redefined the categories and the beta themes into the final version. Finally, the paper was written, describing all categories and themes, and selecting illustrative quotations to complete the thematic analysis workflow. This concluded the last three phases described by Braun and Clarke [27]. The authors changed the working language to English when writing the paper, including translating the quotes.

## Results

After the description of the participants, the results of the analysis are presented with themes and categories.

### Participants

In total, 28 pharmacists participated in the study. In each data collection round, 14 pharmacists participated, totalling 25 women and 3 men. Sixteen of the informants had a bachelor’s degree and twelve had a master’s degree. From the first data collection, 4 of the informants was employed as both pharmacist and as a manager for the pharmacy. The age of the informants was not collected, but the number of years as pharmacists ranged from one to over 30 years, encompassing experience in various pharmacy types in terms of retail settings (Shopping centres, high streets, based in or close to clinic or hospital) and locations (cities, suburban areas, mid-sized towns, and rural areas). All of the participants were employed pharmacists, working for pharmacy chains.

### Themes and categories

**Fig. 1:**
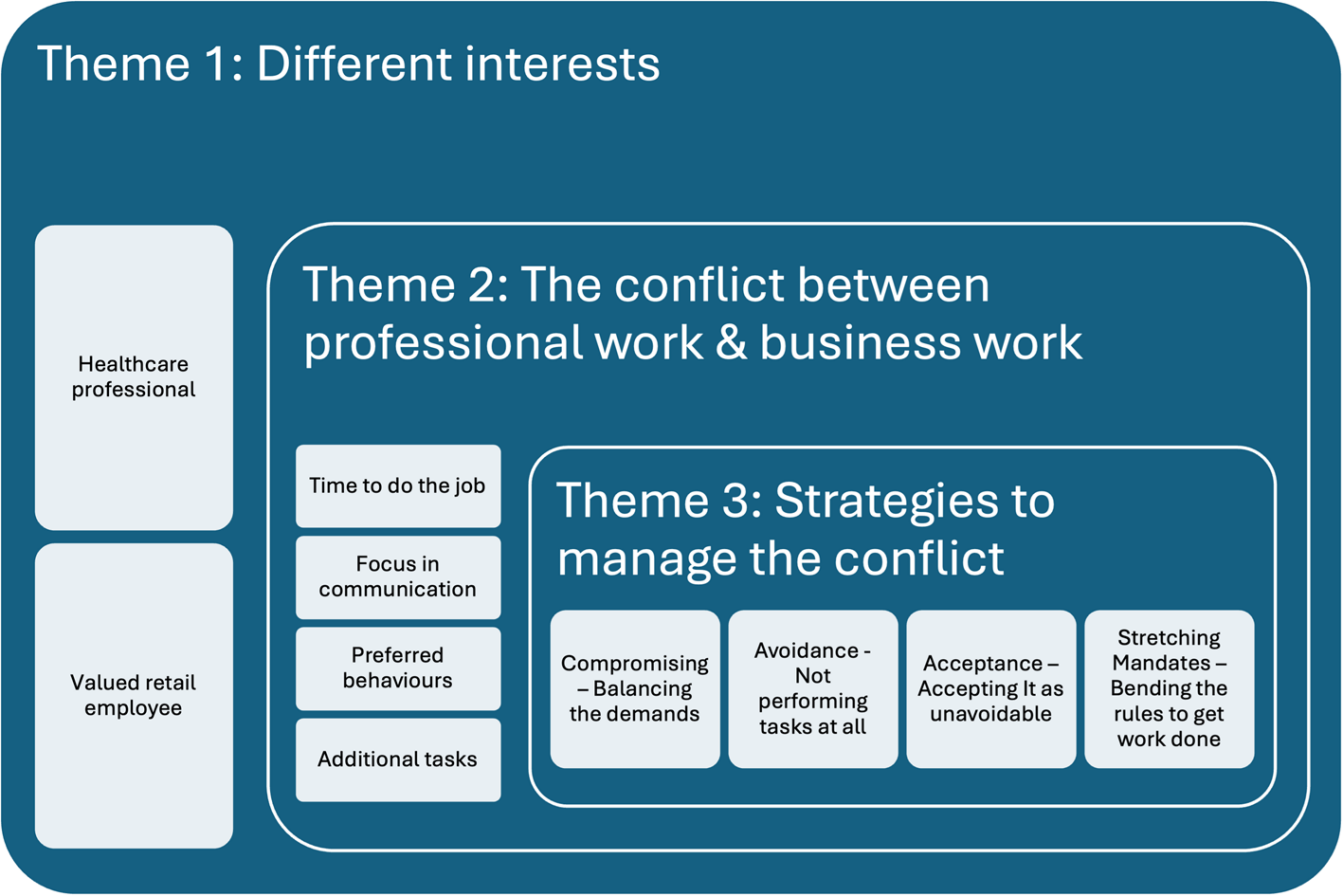
Themes and categories

During the analysis, three themes and ten categories were formulated (Fig. 1). The first theme, *Different interests*, contains the underlying interests that affect the work situation and describes the perceived expectations of the pharmacists. The theme of *The conflict between professional work and business work* is constructed by the categories describing how the two interests’ conflict with each other. The categories in the last theme describe the approaches used to manage conflicts and form the final theme *Strategies to manage the conflicts.*

### Different interests: healthcare professional and valued retail employee

Using terms such as “goals,” “obligations,” “desires,” “expectations,” and “commitments,” the informants depicted two distinct sets of interests: Healthcare professional; performing the professional work (PW) of a licensed pharmacist and Valued retail employee; contributing to profitability through business work (BW).

The presence of the two interests in the daily work was manifested in the ambivalence regarding the terminology used for the recipients of the pharmacists’ services: whether to refer to them as patients or customers. ‘Patient’ was commonly used in pharmacists’ education and in work connected to vocational regulations. From a business perspective, ‘customer’ was perceived as the preferred term, especially by management, and some informants described being corrected when using ‘patient’ in the pharmacy setting. For clarity, the term ‘customer’ will be used in the remainder of the results section.

#### Healthcare professional

The PW interest stemmed from the informants’ perceptions of expectations from various sources and stakeholders, including the healthcare system’s responsibilities as stipulated by the government license, professional ethics, and personal commitment to the vocation. The healthcare system’s expectations were found to align with the pharmacists’ view of what constitutes professional pharmaceutical work, fulfilling professional conduct obligations and delivering high-quality patient care. This included providing advice on safe and effective medication use, dispensing, technical oversight, and medication substitution. Informants noted that PW was not profitable for the pharmacy, necessitating attention to BW as well. Nonetheless, pharmacists predominantly viewed themselves as healthcare providers who also had to perform as retail employees, as illustrated by the following quote:

My role working at the pharmacy is to handle the pharmaceutical aspect. But that’s just not the only thing that is expected of me. I’m supposed to dispense the right product, right strength, right dosage, and everything, check for interactions and such. But then all that other stuff on the side comes in, which is what the company actually makes money from. (Pharmacist #25)

#### Valued retail employee

To be valued as retail employees, pharmacists described obligations and expectations related to their daily work in financial terms, such as contributing to profitability by working efficiently, increasing sales, and reducing costs. Informants felt that the employer emphasized the need for upselling and cost focus for the pharmacy’s financial viability, while also ensuring customer satisfaction to encourage repeat business.

I would say that pharmacies, Swedish pharmacies are essentially stores. They are commercial, profit-driven store establishments that must meet customer demands. (Pharmacist #18)

Pharmacists enhanced profitability primarily by working faster, serving more customers, and avoiding additional staffing expenses.

The employer expects us to be efficient. Obviously, we’ve got to keep things safe for the patients. It’s not black and white. …The employer wants the work done, but quickly, and maybe not dig too deep into things. Like, ‘Let’s pick up the pace a bit’. (Pharmacist #18)

Promoting and selling additional products to customers was also important, as was being a versatile employee to lower costs by performing tasks such as cleaning and restocking. Leaving the prescription counter to assist customers on the shop floor with non-prescription products was also valued in BW.

### The conflict between professional work vs. Business work

The work as a healthcare professional and as a retail employee are assessed using different criteria, due to the representation of different interests, as described in the previous section. Informants described how the expectations surrounding PW and BW led to conflicts in various situations. It was not sufficient to perform core PW tasks; they also needed to focus on the more economically profitable tasks valued by the employer to be considered effective.

…I think it would be impossible to have perfect customer interactions every single time, considering what the employer expects from us, along with all the laws, rules, and the energy and time it takes. Honestly, I don’t think it’s possible for anyone, no matter how skilled or meticulous they are. (Pharmacist #17)

There was an expectation to offer and sell additional products to every customer and to serve as many customers as possible. Conversely, informants argued that service quality should be the focus, especially concerning PW. This conflict often became challenging to manage due to time constraints, which limited or hindered pharmacists’ ability to adhere to both interests simultaneously. Pharmacists were forced to decide which interest to prioritize in the situation. The following four categories labelled ‘The Time to Do the Job,’ ‘The Focus in Communication,’ ‘Preferred Behaviour,’ and ‘Tasks to Perform at the Workplace’ describe situations and decision points where the CoI influences the work.

#### Time to do the job

Firstly, the informants described that a short waiting time for each customer was a priority in their BW, and there were defined targets for this waiting time. The time spent with each customer was expected to be as brief as possible to minimize the waiting time for others. Consequently, there was an expectation from the employer, customers, and sometimes colleagues, to “push forward” new customers quickly instead of conducting high-quality PW.

For those customers who require it, I would like to feel that I can focus more on providing advice, perhaps sit down with the customer, open the package, go through the leaflet, or show them, in a calm and relaxed manner, how the inhaler works, or whatever it may be, and demonstrate more practically. And not have to feel that I need to rush to the next customer. (Pharmacist #27)

The above was experienced as a CoI since, in PW, each customer and the dispensing of prescriptions have several aspects that need to be controlled and performed. For example, the correctness of the prescription needed to be verified in terms of it being the correct medication and the right dosage, potential problems with interactions between medicines needed to be checked, and the pharmacist needed to ensure the customer’s understanding of the treatment and the use of the medication. At the same time, many pharmacies had a system for displaying how long customers had been waiting, which signalled when it was considered too long, and this was perceived as stressful. The pharmacists described that spending too long a time with a customer could also affect their colleague, who had to work even faster, which added to the stress.

#### Focus in communication

Besides keeping the waiting time short, the informants described how BW is emphasized in the communication related to the overall management of the pharmacies, at the expense of PW. The priority of BW was also reflected in the KPIs (sales), in management communication, and in the staff meetings, which had been renamed to “sales meetings” in many pharmacies.

One of the key performance indicators common in pharmacies, and described as an important one, was the level of “combination receipts.” These receipts indicate how well the pharmacists succeeded in selling additional products by measuring the number of customers who both had a prescription filled and left the pharmacy with at least one other product.

Just so you know, if we manage to get both a prescription item and a non-prescription item on the same receipt, it counts more favourably than if the customer purchases them separately, at least in terms of how the employer analyses the financials. (Pharmacist #20)

The way for pharmacists to improve their performance concerning combination receipts was to inform the customer of different promotions and marketing activities or in other ways selling additional products. These sales activities were viewed as being at the expense of PW. The informants described how BW was the focus of performance reviews and the yearly salary discussions, while PW was seldom discussed during these meetings.

Sales took time away from PW in customer dialogue, but it was also experienced as devaluing the profession. Informants explained how being viewed as a salesperson could influence their ability to provide professional advice and vice versa.

I find it problematic that roles become conflated, and I believe that someone who is not a pharmacist may not perceive this. After all, it is a licensed profession, a conscience-driven occupation. Customers should be able to trust that what I say is objective and true when I discuss medications. In sales, there is no objective truth. (Pharmacist #1)

Many examples were given where the customer showed limited understanding of the pharmacist’s role and competence and was viewed more as a store employee who should just hand out the medicine without asking any questions.

Some informants described a similar reaction when they, as pharmacists, gave valid advice, but it was disregarded by the customer as only an attempt to sell something.

#### Preferred behaviours

If something is unclear with the prescription that needed to be discussed with the prescriber, many informants consider it to be an important part of their professional work. Some employers discourage pharmacists from contacting the prescriber and instead advise the customer to check with their doctor themselves and then come back. Shifting the responsibility to the customer could have potentially harmful consequences, as one informant described:

…many times, the customer has left and contacted the doctor directly, but often, the customer comes back after weeks, and then you see on the list that the person hasn’t even picked up their medication. It was a really important medication. ‘Why didn’t you pick it up?’ ‘Well, I was supposed to contact the doctor, but I couldn’t reach them, so I just didn’t bother’. (Pharmacist #22)

Some employers also provided a set of preferred behaviours concerning interactions with customers, including offering additional products. Informants described instructions, sometimes in the form of a script that was expected to be followed and could include how employees should greet the customer upon entering, making eye contact, etc. The behaviours that the employers advocated were perceived as mostly directed towards BW, even if they could include tasks associated with PW. Several informants described how these behaviours were enforced by using fake customers, or Mystery Shoppers as they are called, who pretended to be customers but were appointed by a third party, at the employer’s initiative, to collect data based on a checklist grading different aspects of work, mostly concerning waiting time, eye contact, wording in the communication, and if the pharmacists offered additional products.

In summary, to be viewed as a good employee, you are expected to follow the proposed way of behaving, but the mandated behaviours in customer meetings were felt in varying ways as unnatural, and some informants felt it even undermined their professional role.

“There’s a whole structure, a template, on how to interact with customers, and every little step is scored, which I find almost ridiculous. It’s as if they’re trying to eliminate all common sense from dealing with people.” (Pharmacist #28).

#### Additional tasks

Fourthly, there were some tasks that an employee needed to handle, as part of a pharmacy being a retail store, that were viewed as being far from the PW they signed up to do. These tasks could include stocking products, cleaning, and working with sales as a cashier in the self-care section, or “out-there” as it was commonly called (i.e., the part of the pharmacies where the non-prescribed assortment was located, in contrast to “behind the counter” where the pharmacist’s primary work was done). These extra activities took time away from customer dialogue.

“…pharmacists who unpack items… When we’re so few pharmacists at the pharmacies, and on top of that… dust shelves? To me, it’s incomprehensible that we should spend our time on that when we’re needed for our customers, and that’s where our expertise lies.” (Pharmacist #25).

### Strategies to manage the conflict

Pharmacists often found themselves in the position of being both a professional and a retail employee. To handle this situation, they employed various strategies. Four different categories of strategies to manage this conflict were identified in the analysis: compromise, stretching the mandate, avoidance, and acceptance.

#### Compromise – balancing the demands

A common strategy widely used was to compromise; to find ways to be both a responsible healthcare professional and a valued retail employee. One example was to sell with consideration, using professional knowledge to promote additional products directly linked to the treatment. This was framed more as professional advice, even if it simultaneously added to profitability. A common example of this strategy was to offer saliva-enhancing products to customers who had been prescribed medication that could cause mouth dryness, as this condition can damage the teeth. This was described as a way of doing what was expected without compromising one’s beliefs. It was sales, but it was based on knowledge and the well-being of the patient.

“So, I’m focusing on the pharmaceutical side of things, and maybe I’ll find something else along the way. You know, with us, if we find the product of the week a bit tricky, we just pick something we think fits well… Like, we use a lot of saliva stimulators because everyone can benefit from that.” (Pharmacist #15).

Another way of compromising was to ask a concluding question, after the professional tasks were done, that allowed the customer to ask the pharmacist about additional products, instead of actively recommending or promoting products.

“I want my boss to see that I’m a good employee, so I try to do both as well as I can. Not to the extent of pushing something onto someone, but just asking every customer, ‘Is there anything else you need today while you’re here?’” (Pharmacist #21).

Compromising was also used to manage time, by choosing whom to offer additional products to, and whom not to. If a customer had a more complicated prescription or a lot of different medicines, the pharmacist skipped it but added additional products to customers with less complicated prescriptions. They saved some time by not doing it for everyone.

#### Avoidance – not performing tasks at all

Informants described that they sometimes just avoided performing tasks, mainly to save time and keep lines short. One example of this was to keep the customer dialogue as short as possible, such as prioritizing the most important duties to fulfil legal obligations in their professional work and avoiding asking open-ended questions that risked prolonging the meeting to do good business work.

“I try to use my (…) ability to make quick judgments even there, to try and make an informed, as best as I can at the moment, decision about how important it is to skip some type of question or discussion with the customer.” (Pharmacist #17).

Another example was that when there was no customer in the line for prescribed medicine, the pharmacists were expected to go to the self-care area to help sell products there, but some avoided it and stayed behind the counter. Another example was, that instead of contacting the prescriber as part of the professional work, they told the customer to do it themselves to save their own time. Refusing to take part in some sales activities and doing the “pin-statistics” was a fourth example; some did it openly, while others did it more in secret.

“I sell if it’s needed. If it’s not needed, then I don’t sell. It means if I lose my job over it, then I’ll walk away.” (Pharmacist #22).

Another informant described that he/she did not participate in some of the sale activities and did not present how many products they sold. When asked what would happen if being asked about it by the manager, the answer was:

“No, but I’m supposed to present it, so then I’m lying.” (Pharmacist #28).

#### Acceptance – accepting it as unavoidable

Some informants’ descriptions of how they managed the conflict between professional work and business work were descriptions of acceptance. Even if some did not want to perform some tasks, they did it anyway. Even if they recognized that the circumstances for doing the work were not ideal, the pharmacists explained it was necessary to participate in business work, with the motivation that if the pharmacy was not profitable, it would be closed.

“I know what’s expected of me at work, and if that doesn’t fit, maybe I should work somewhere else.” (Pharmacist #21).

#### Stretching mandates – bending the rules to get work done

The time limitation made it difficult to follow all rules and regulations, and pharmacists sometimes perceived that they were expected to change how they were doing their professional work to adhere to more business needs. By stretching the mandate, either in the professional work or in the business work, pharmacists saved time. In professional work, the informant described how they could change the package size or combine the strength of the tablets to reach the prescribed dosage to help the customer fill the prescription at once when out of stock. This could also speed up the work, omitting the step of clearing it with the prescriber, which was perceived as what the regulation demanded.

“There are rules that the pharmacist must make absolutely sure the patient knows how to take their medications. But in practice, you can just ask, ‘Do you know how to take these?’ and if they say ‘yes,’ that’s it.” (Pharmacist #18).

Several examples were given on how the informants bent the rules to save time and to get the customers what they needed. It was often described as something necessary to do, while others said that they would never do it because one should not break the rules.

## Discussion

In the work of pharmacists in Swedish community pharmacies, incompatible interests exist, which call for different actions and decisions and inline with the aim of this paper, the ambition is to explore this conflict, and how it is managed.

### The conflict between being a healthcare professional and viewed as a valued retail employee

In line with earlier research [5, 8, 29] our study reveals that pharmacists do experience conflicts between upholding professional conduct (i.e., doing PW) versus being good employees within a retail company (i.e., doing BW), primarily due to constrained time. Another study showed that lack of time caused ethical dilemmas for pharmacists [30].The perception that there is an expectation from the employer to work fast and also to find time to offer additional products to customers, is also in line with what other studies show [31]. Pharmacists must perform tasks ensuring they are perceived as both professional healthcare workers and valued retail employees. Neither type of work could be neglected without cost: but there was insufficient time for both. Neglecting PW could harm not only the pharmacist’s ambitions as a healthcare professional and lead to loss of license but also potentially harm customers. On the other hand, neglecting business work BW could jeopardize a pharmacist’s financial situation due to lack of salary increases or career opportunities and ultimately affect job security, as it is, according to Hibberth et al. [32], a commitment as a pharmacist to be a part of generating profit.

The study makes it evident that pharmacists experience their professional interests as influenced by secondary interests, which is in line with how Thompson defines conflicts of interest [19]. Komesaroff et al. [14] describe that CoI can encompass both financial and non-financial interests. In our study, PW was primarily assessed by pharmacists’ desire to deliver quality healthcare—considered non-financial—while BW was assessed by their desire to be seen as good employees—understood as mainly non-financial but also involving financial aspects. Financial CoIs are typically straightforward both in detection and assessment, while a non-financial CoI is more difficult to see and assess. Komesaroff et al. [14] describe three criteria to assess the importance or relevance of non-financial interests: it must be relevant to the situation; could have a considerable effect; and have a certain permanence, meaning it cannot easily be altered by just talking or reflecting about it. First, in our study, we understand the desires and the obligations of the interests are very relevant to the pharmacists in their day-to-day work, and they need to make conscious decisions on how to act. The actions in the PW that fulfill the desire to be a professional healthcare provider are not always equal to actions assigned to being a valued employee [cf. 5]. Second, depending on the decisions made, the action, or lack of action, can lead to severe effects on the outcome. For example, if the sole focus is on BW, the quality of the service regarding both the dispensing of the medicine and the knowledge about the proper usage of it could be affected [cf. 4]. If instead, only PW was prioritized, it could increase the waiting time, result in fewer customers and less sales, as well as the pharmacist could be regarded as a less contributing employee, that does not take responsibility for the financial gain and stability of the business. Finally, the non-financial interests and the following conflict are not temporary, it is a result of the current circumstances that govern the pharmacy market and will not go away by discussing it or by introducing policies [cf. 14]. The way work is organized, where pharmacists manage their daily practice independently [e.g., 9], results in each pharmacist handling the CoI by themselves. Our study shows that even if the employer acknowledges the importance and the quality of the PW, the pharmacists experience a conflict that they have to manage in order to be viewed as valued employees as well as to deliver the work that they, as professionals, both desire and view as obligated. Thus, what this study contributes by using the theory of CoI, is problematizing the work situation of community pharmacists, and showing that it is affecting everyday work and decisions.

### Strategies to manage the conflict

Earlier research has identified the dual expectations affecting community pharmacists [4, 5, 12, 26] while this study also examines the strategies pharmacists use to manage them. To manage the conflict between doing PW and BW, pharmacists in this study engaged in four strategies: compromising – balancing the demands; stretching mandates – bending the rules to get work done; avoidance – not performing tasks at all; and accepting – accepting it as unavoidable. In both the strategies of compromising and stretching the mandate, cognitive and task-crafting approaches seemed to interplay with each other. In the strategy of compromising – balancing the demands, the cognitive aspects of crafting related to mental strategies of justifying ways to be both a responsible healthcare professional and a valued retail employee. By choosing to, to some extent, adhere to conflicting interests through mental processes of compromising, the pharmacists in the study found ways of altering their performance (i.e. task crafting) based on how they thought about the work. Cognitive crafting preceded, in other words, and motivated what tasks to prioritize. The pharmacists applying this strategy seemed to be eager to in different ways compromise to perform both PW and BW in satisfactory ways. Compromising does not mean that it is the objectively best action to take for the customer or the employer, but it shows the importance of the ambition to be both a healthcare professional and a valued retail employee.

In the strategy of stretching the mandate – bending the rules to get work done, pharmacists increased their perceptions of meaningful work by permitting themselves to make decisions more or less against the rules, based on their professional knowledge, to serve the needs of the customer but also to save time, and thereby accommodate the ambition to be a valued retail employee. Not adhering to the rules could be viewed as rebelling against the lack of mandate and autonomy in the vocation, but it also implies that saving time becomes very important. The strategy of avoiding – not performing tasks at all is defined as task-oriented based on the definition provided by Wrzesniewski and Dutton [16]. Avoiding tasks becomes another way of saving time, or to prioritize other tasks, mainly to the benefit of the PW. It could be consequences of avoiding doing tasks that one is expected to do, but applying this strategy implies that the underlying motives make it worth the risk. The final strategy of acceptance – accepting it as unavoidable could be seen as an example of cognitive crafting The strategy included accepting to perform tasks that were not perceived as part of their job, for the sake of being viewed as a valued employee. In this strategy, pharmacists cognitively accepted parts of the BW as a condition for performing PW in a pharmacy.

Thus, when choosing strategies for how to deal with the CoI it seems that the PW often was prioritized, which is in line with previous research showing that employees in pharmacies perceive a stronger connection to and identification with their vocation compared to the company [9, 12]. It also seems that pharmacists sometimes perform BW that is cognitively motivated by how it contributes to PW. An example of this is when additional sales are redefined by some as a health promotion activity targeting side effects of medication or other issues closely connected to the patient’s prescription. To note, none of the identified strategies could be defined as relational crafting. This can be seen as surprising since a previous study shows that being a part of a team at the pharmacy was important and something that employees valued [9]. This could be explained by the independent way of working with limited involvement of colleagues or the supervisor. That work at pharmacies is conducted independently to a large extent, confirms what other studies have indicated [4, 5, 9]. However, a consequence is also that individual pharmacists must handle the conflict themselves as well, which could lead to ethical stress and strain.

The pharmacy market is governed by granting businesses permission to trade with pharmaceuticals and thereby becoming a part of, or at least an extension of the healthcare system [1, 18]. One of the obligations to keep the permission is to employ pharmacists with individual licenses to dispense prescribed medicine to perform the work [1]. The individual license requires the pharmacist to work for the best interest of the healthcare system and thus the patient, while as an employee, there is an expectation, or obligation, to act in the best interest of the employer. The business, on the other hand, has an overarching objective, or more precisely, a demand, to deliver financial value to its owner. These differences are the foundation of the different interests that affect the work situation of employed pharmacists.

This study has three main contributions. First, by problematizing the work of community pharmacists through the lens of conflict of interest, it becomes apparent that pharmacists need to make trade-offs in their day-to-day work. In the pharmacy context, the two interests are perceived as legitimate and not per definition conflicting, while in the pharmacists’ daily work, this study shows that they are conflicting, especially when considering the limited time available with each customer. Second, this study presents four strategies that pharmacists engage to manage the conflict of interest that occurs when aspiring to be both a professional healthcare worker and a valued retail employee. Third, the study reveals that the discussion within the workplace regarding the conflict and the actions that pharmacists can take to manage the potential negative consequences of it is not particularly vibrant. This could form part of future developmental efforts; by acknowledging and discussing these issues, the responsibility could be collectively shared at the workplace and not fall solely on the individual pharmacists. However, merely talking about it does not resolve the CoI, but it could potentially be a starting point for exploring solutions. One thing is certain: even with increased awareness of the issue, the conflict persists and may have the potential to decrease the trust in the vocation and the individual [12, 33], which our study also indicates.

## Method considerations/limitations

The study is based on two different data collections, which differ in terms of time, interview guide, and selection criteria. The value of using a second data collection to enhance the understanding of the conflict and strategies for managing them was assessed to exceed potential disadvantages.

With the second data collection, the study was also strengthened by adding informants with experience from various chains, and types of community pharmacies, both in terms of retail settings and geographical locations. The intention was to ensure that the informants represented a variety of experiences to provide us with the opportunity to explore the perceptions of employed pharmacists more broadly.

Most of the quotes used in the manuscript derived from the second phase of the data collection, most certainty due to the more focused questions which opened for more elaborating answers concerning the conflicts of interest compared to the first phase where the questions were more comprehensive.

None of the authors are pharmacists, but one of the authors, KH, had previously worked at the head office of a pharmacy company. To limit any potential bias due to preconceived notion etc., the authors discussed the data, the analysis and the results, and finally wrote the manuscript together.

## Conclusion

Pharmacists predominantly view themselves as healthcare providers, but as employees in a retail operation, they are also expected to adhere to business needs. For pharmacists, it becomes a juggling act to perform the work as professional healthcare workers and, at the same time, be viewed as valuable retail employees, which creates a conflict of interest. Due to the limited time that pharmacists perceive they can assign to each customer, decisions on what activity should be performed become necessary, resulting in a prioritization of either professional work or business work. Pharmacists need to reshape parts of their work to meet these demands, which could be explained by the individual process of job crafting. However, even if job crafting is done individually, a pattern in the outcome was identified, which is viewed as strategies to mitigate the conflict. By viewing community pharmacists’ work from a conflict of interest perspective, it becomes evident that the responsibility to navigate the situation falls on the individual pharmacist, while the consequences of their actions could affect the healthcare system, customers, colleagues, and the employer.

When discussing pharmacist practice, profession, or role in the healthcare system, the conflict of interest embedded in the practice needs to be taken into consideration. It could affect the efficiency of the pharmacist’s work, as well as the outcome. In addition, it could also impact the ability of pharmacists to take on additional tasks and services, as well as the potential to attract prospective future pharmacists.

## Data Availability

Data are not publicly available due to participants not having consented to public availability.
